# Quality of medicines in southern Togo: Investigation of antibiotics and of medicines for non-communicable diseases from pharmacies and informal vendors

**DOI:** 10.1371/journal.pone.0207911

**Published:** 2018-11-29

**Authors:** Simon Schäfermann, Emmanuel Wemakor, Cathrin Hauk, Lutz Heide

**Affiliations:** Pharmaceutical Institute, Eberhard Karls University Tuebingen, Tuebingen, Germany; Paediatric Centre of Excellence, ZAMBIA

## Abstract

Substandard and falsified medicines represent a serious threat for public health and patient safety. Especially in low and middle-income countries, the prevalence of substandard and falsified medicines is reportedly high. However, reliable information on the prevalence of poor-quality medicines is scarce. In this study, 12 essential medicines, including antibiotics, antidiabetics, cardiac drugs and antiasthmatic drugs, were collected from six informal vendors and six licensed pharmacies in the southern part of Togo (regions Maritime and Plateaux). A mystery shopper approach was used in both types of outlets. In total, 64 samples were collected from licensed pharmacies and 30 from informal vendors. Both availability of medicines and prices of medicines were higher in licensed pharmacies than in informal vendors. 92 medicine samples were analyzed by visual examination, followed by chemical analysis for the content and for the dissolution of the active pharmaceutical ingredients according to the respective monographs of the United States Pharmacopoeia. 7 samples (8%) did not comply with the pharmacopoeial specifications, and one sample (1%) showed even extreme deviations. None of the samples was obviously falsified. However, one sample of amoxicillin capsules contained only 47% of the declared content of the active pharmaceutical ingredient, indicating that it may represent amoxicillin capsules 250 mg, rather than 500mg as declared on the label. Medicines stated to originate from Asia (i.e. mainly from India and China) showed a significantly higher proportion (24%) of non-compliant samples than those from Africa and Europe (4%, p = 0.007). High failure rates were observed in medicines both from informal vendors (13%) and from licensed pharmacies (5%), but the difference between both groups was not statistically significant (p = 0.152). The observed high prevalence of substandard medicines requires action from regulatory authorities and health care providers. Testing of selected samples for related substances indicated that inappropriate transport and storage conditions may have been an important cause for substandard quality.

## Introduction

Access to essential medicines has been included in the Millennium Development Goals (MDG) of the United Nations and is now included in the Sustainable Development Goals as Goal No. 3.8 [1]. It comprises access to medicines for both non-communicable diseases (NCD) and communicable diseases [2, 3]. Access to medicines in developing countries has improved in the past decades [4, 5]. However, at the same time the occurrence of substandard and falsified medicines has been reported frequently and has even been addressed as a “pandemic” by some authors [6]. A recent literature survey by the World Health Organization, estimated that in low and middle-income countries 10.5% of the medicines are substandard or falsified [7]. Several studies and reviews tried to estimate the prevalence for specific countries and regions and for different classes of therapeutics. A systematic review by Almuzaini et al. included eight studies conducted in sub-Saharan Africa which reported prevalence for substandard or counterfeit medicines ranging from 12.2 to 46% [8]. A meta-analysis of 21 surveys in sub-Saharan Africa concluded that 35% of antimalarial medicines had failed chemical analysis, and 20% had been classified as falsified [9]. However, other studies reported a much lower prevalence of falsified medicines. In several studies by the ACT Consortium Drug Quality Program (ACTcDQP), only 98 (0.97%) out of 10,079 antimalarial medicine samples from six developing countries were reported to be falsified [10]. An investigation by the World Health Organization in six countries of sub-Saharan Africa found only 2 (0.2%) out of 935 antimalarial medicine samples in which a stated active ingredient was missing entirely [11]. Several authors emphasize that there is still a severe lack of reliable data on the prevalence of substandard and falsified medicines [8]. The report of the Lancet Commission on Essential Medicines also concluded that so far, the true extent of this problem remains unknown [12]. Also the above-mentioned literature survey by the WHO discusses the problem of the strong heterogeneity of the available studies [7].

Until recently, the lack of internationally agreed definitions of substandard and falsified medicines further complicated the comparison of data on their prevalence. This situation has recently been remedied by the World Health Organization [13]. The misleading term”counterfeit medicines” as well as the provisional term “substandard/spurious/falsely labelled/falsified/counterfeit (SSFFC) medical products” have been replaced by “substandard and falsified medical products” [13, 14]. Notably, ”substandard” and “falsified” are now defined as mutually exclusive classifications. Falsified medical products are “deliberately/fraudulently misrepresenting their identity, composition or source”, while substandard medical products fail to meet their quality standards or specifications for other reasons than deliberate intent, e.g. due to unintentional shortcomings in the manufacturing process, or due to degradation caused by inappropriate storage conditions. Differentiation between falsified and substandard medicines therefore requires knowledge or clues of the (honest or fraudulent) intentions of the manufacturer, and is not possible on the basis of chemical analysis alone [15].

Most published studies on substandard and falsified medicines have focused on anti-malarials, some on antibiotics and antivirals, but data on medicines for non-communicable-diseases are rare [16, 17]. This is in contrast to the importance of non-communicable diseases also in developing countries. The last report published by the MDGs Gap Task Force in 2015 noted that up to 80% of the deaths from non-communicable-diseases world-wide occur in low-and middle-income countries [18, 19].

The institutional capacity and the resources to monitor the quality of the pharmaceuticals are diverse in between different African countries. Several East-African countries have successfully strengthened their regulatory authorities [20], other African countries still struggle with the implementation of a regulated supply of medicines. For this and for other reasons, the prevalence of substandard and falsified medicines is very different in between African countries [10, 11]. Tabernero et al. noted that there is a severe lack of knowledge on the prevalence of substandard and falsified medicines in quite a number of African developing countries, including Togo [21].

Only very few studies investigated substandard and falsified medicines in the Republic of Togo so far. In 2014, an evaluation of the quality of artemisinin-based antimalarials was conducted in Ghana and Togo. It reported that 83.7% of artemisinin based combination therapies and 57.9% of the artemisinin-based monotherapies failed to comply with International Pharmacopoeia requirements due to insufficient content of the active pharmaceutical ingredient (API) [22]. The recently published quality evaluation of cardiac medicines in ten countries of Africa [17] also included 100 samples deriving from Togo. It concluded that 9% of the samples deriving from Togo where poor quality drugs. Also a study on the quality of veterinary medicines was carried out in the northern part of Togo [23].

The objective of the present study was to contribute to the knowledge about the prevalence of substandard and falsified medicine in the Republic of Togo, including both anti-infective medicines and medicines for non-communicable diseases. Medicines were sampled from the private sector, i.e. licensed pharmacies, and informal vendors, in several towns in the southern part of Togo.

## Methods

### Study design

Collection of the samples took place in February 2017. The study protocol was based on the guidelines on the conduct of surveys of the quality of medicines, published by the WHO in 2016 [24] and the MEDQUARG guidelines [25]. Seven antibiotics and five medicines against non-communicable diseases were included in this study, all of them contained in the list of essential medicines of the Republic of Togo [26]. These 12 medicines were solid oral dosage forms of amoxicillin, amoxicillin/ clavulanic acid, sulfamethoxazole/trimethoprim, ciprofloxacin, phenoxymethylpenicillin (penicillin V), metronidazole, doxycycline, metformin, atenolol, hydrochlorothiazide, furosemide and salbutamol (albuterol.) For each medicine, a preferred strength and dosage form (tablet or capsule) was defined which the local investigator asked for at every sampling site. If this was not available, another strength or another solid oral dosage form was collected.

The samples were collected in the southern part of Togo, in the regions Maritime and Plateaux. In Lomé the local investigator asked several citizens for well stocked informal drug vendors in the south of Togo. Five such vendors were named in the region Maritime located in Lomé Centre, in the suburbs Agoe-nyvie and Agoe-laogope, as well as in the towns Tsévié and Tabligbo, 30 km north and 75 km northeast of Lomé, respectively. Furthermore, one vendor was named located in the town of Kpalimé, 120km northwest of Lomé and close to the border to Ghana, in the region Plateaux. S1 Fig (Supporting Information) shows a map of these locations. The informal vendors operated in small shops located away from the main shopping roads. Such non-accredited medicine stores in Togo and neighboring countries have been described already in earlier studies [22, 23]. While visiting each of the six chosen informal drug outlets, the investigator identified the geographically nearest licensed pharmacy. A list of licensed pharmacies in Togo is available in the internet [27], and indeed all six pharmacies named by the local citizens were found in this list, but none of the informal vendors. Licensed pharmacies operated in premises with good professional appearance, located at major shopping roads. In both types of sampling sites the investigator acted as a customer and purchased a quantity of 100 tablets or capsules for each of the 12 medicines, if available. If the vendor asked for the purpose of the purchase, the investigator stated that these medicines were intended for use in a local medical facility operated by a relative. If the quantity of 100 tablets or capsules per medicine was not available, a smaller quantity was purchased, but not less than 30 tablets or capsules to ensure a sufficient amount for chemical analysis. At each of the twelve sampling sites, prices and quantities of purchased medicines were recorded. Each sample was collected and stored in the original primary and secondary packaging if possible. If no primary or secondary packaging was available, the samples were stored in light protective screw-cap bottles. An adhesive label with a unique sample number was attached to the primary or secondary packaging of each sample. The samples were stored at a cool and dry place and transported to the University of Tuebingen, Germany, within three weeks after collection. There, all medicines were stored at 21°C in an air-conditioned room until analysis.

### Sample size calculation

The sample size calculation was based on the hypothesis that the proportion of out-of-specification medicines would be higher in medicines from informal vendors than in medicines from licensed pharmacies. Estimating the proportions to be 10% in medicines from licensed pharmacies and 40% in medicines from informal vendors, the sample size required to observe a significant difference between these group with 95% confidence and a power of 80% resulted as 29 samples per group, using the following formula:

$$\text{n}={\left({\text{Z}}_{\alpha /2}+Z_{\beta }\right)}^{2}*\left({\text{p}}_{1}\left(1-{\text{p}}_{1}\right)+{\text{p}}_{2}\left(1-{\text{p}}_{2}\right)\right)/{\left({\text{p}}_{1}-{\text{p}}_{2}\right)}^{2},$$

where Z_α/2_ is the critical value of the Normal distribution at α/2 (e.g. for a confidence level of 95%, α is 0.05 and the critical value is 1.96), Z_β_ is the critical value of the Normal distribution at β (e.g. for a power of 80%, β is 0.2 and the critical value is 0.84) and p_1_ and p_2_ are the expected sample proportions of the two groups [28].

We decided to attempt the collection of 12 medicines from 6 facilities, i.e. 72 samples, from each group, in order to allow for a contingency since not all medicines were expected to be available in each of the sampling sites.

### Medicine quality analysis

Analysis was performed at the Pharmaceutical Institute of Tuebingen University. Prior to chemical analysis, the packaging of each sample was visually examined. Sample number, brand name, type of medicine (originator, branded generic or generic), batch number, manufacturing date, expiry date, name of marketing authorization holder (MAH), name of manufacturer, international non-proprietary names (INN) of the active pharmaceutical ingredients (APIs), dosage form, strength, type of packaging material or container (primary and/or secondary packaging), presence of a leaflet for patients, and price per dosage form were recorded on a standardized form. Digital photos showing the tablets or capsules, the primary packaging, the leaflet, the secondary packaging and the sample number were taken and archived.

Chemical analysis was carried out according to the methods specified in the monographs of the United States Pharmacopoeia 2016 (USP 39) for the respective dosage forms for each of the 12 medicines. The chemical quality assessment included the determination of identity, assay (content of API) and dissolution (proportion of API dissolved from the dosage form over time). Following the respective monographs of the USP 39, the assay (quantification of the content of the API) was carried out by HPLC for all investigated medicines, and dissolution of the API was quantified by HPLC for all investigated medicines except metformin, which was quantified by UV spectroscopy.

Validation of the assay procedures according to USP using medicines purchased in Germany showed good reproducibility of the results. In contrast we initially noticed high variability of the assay results from some of the samples collected in Africa, e.g. of amoxicillin/clavulanic acid tablets. Further investigation showed that a more thorough mechanical disintegration of these tablets was required in the sample preparation, to yield complete and reproducible detection of the API content. USP specifies these mechanical disintegration procedures only in general terms. (“dissolve not less than 10 tablets in water with the aid of mechanical stirring”). According to our observations, tablets which have been stored under tropical climates require thorough mechanical disintegration in order to achieve correct assay results, and the same observation has recently been reported by Mufusama *et al*. [29].

One sample of amoxicillin (QEW067) was found to contain only 47% of the declared API content; it was tested for content uniformity according to the method for of USP 39, determining the contents of 10 tablets individually. Furthermore, for two samples of amoxicillin/ clavulanic acid and one sample of hydrochlorothiazide tablets, content uniformity was investigated by analyzing 10 tablets individually, after sample preparation with a Branson 250 Sonifier (Emerson Industrial Automation, St. Louis, MO, USA) pulsing at 70% power, in an interval of 10 seconds with 10 seconds intermission for a total duration of three minutes using the same solvent as stated in the USP39 monograph. HPLC analysis according to USP showed a high uniformity of the content (QEW002, amoxicillin SD = 0.70%, clavulanic acid SD = 2.46%; QEW041, amoxicillin SD = 2.63%; clavulanic acid SD = 2.44%; QEW074, hydrochlorothiazide SD = 1.70%, respectively). The assay value reported in this study for these three samples is therefore the average of the 10 individual measurements for each sample.

All methods were validated according to USP instructions for system suitability and the Q2 Guideline of the International Council for Harmonization [30]. HPLC analysis was carried out using an Agilent 1100 HPLC (Agilent Technologies, Santa Clara, CA, USA) and the columns, mobile phases and UV-detection wavelengths specified in the USP 39. UV spectroscopy was carried out using a Perkin-Elmer Lambda 125 UV spectrophotometer (Perkin-Elmer, Waltham, Massachusetts, USA). Dissolution testing was performed using a PTWS 610 Dissolution Testing apparatus (Pharma Test Apparatebau AG, Hainburg, Germany).

### Definition of compliance of samples with specifications

The USP 39 criteria of the respective monographs were followed in assessing compliance or non-compliance of the investigated samples. The limits for compliance stated by the USP 39 are different for different APIs and are summarized in Table 1. Samples falling outside of these limits were considered as non-compliant.

**Table 1:** Limits for compliance according to United States Pharmacopoeia 39 [% of declared content].

API	assay	dissolution
Amoxicillin	90–120	75
Amoxicillin / clavulanic acid	90–120	85/80
Penicillin V	90–120	75
Ciprofloxacin	90–110	80
Sulfmethoxazole / trimethoprim	93–107	70/70
Metronidazole	90–110	85
Doxycycline	90–120	80
Metformin	95–105	70
Atenolol	90–110	80
Hydrochlorothiazide	90–110	60
Furosemid	90–110	80
Salbutamol	90–110	80

As proposed by a study published by the WHO in 2011 [11], the non-compliant samples were further divided into those showing only moderate deviations from the USP 39 criteria, and those showing extreme deviations. As suggested by the mentioned WHO study, extreme deviation was defined as the content of API deviating more than 20% from the declared content and/or the average dissolution of the tested units falling more than 25% below the pharmacopoeial limit (i.e. below the pharmacopoeial Q-value minus 25%).

For the combined results, a sample was rated as “non-compliant” if either assay or dissolution or both tests had failed.

### Testing for related substances

Selected samples containing amoxicillin were tested for related substances following the method stated in the USP 39 monograph for amoxicillin trihydrate. HPLC peaks of possible degradation products observed in these samples were compared to peaks appearing in reference tablets that had been subjected to forced degradation at 80°C in a drying oven for 4 days [31]. As explained above, in this study, the classification as compliant or non-compliant was based only on identity, assay and dissolution without considering the absence or presence of related substances, since only few samples were tested for related substances.

### Calculation of medicine prices

The prices of all samples were recorded in local currency (CFA francs). The price per dosage form was calculated in US $ with the exchange rate [CFA] to [US $] of 01.02.2017 (1 CFA = 0.00163428 $). As suggested by the WHO/HAI manual on measuring medicines prices [32] a Median Price Ratio (MPR) was calculated, i.e. the ratio of the observed median price of a medicine to an international reference price. As recommended by the WHO/HAI manual, the median supplier price from the MSH 2015 international medical products price guide [33] was chosen as international reference price. If medicines of different strengths were collected, median prices and MPRs were calculated individually for each strength.

### Statistical analysis

Statistical analyses were performed using MedCalc (MedCalc Software, Ostend, Belgium) [34, 35].Comparisons of proportions were evaluated by the "N-1" Chi-squared test as recommended by Campbell (2007) and Richardson (2011) [36, 37]. Confidence intervals were calculated as the "exact" Clopper-Pearson confidence interval for the observed proportion [38, 39]. Prices of medicines were compared to an international reference price (see above), and differences of the resulting price ratios were evaluated using the Kruskal-Wallis test.

## Results

### Overview of collected samples

In this study 12 different medicines were collected from six licensed pharmacies and six informal vendors, resulting in a theoretical total of 144 samples. Because not all sampling sites had all medicines in stock, we were able to purchase a total of 89 samples. Visual examination showed that two samples contained blisters from two different manufacturers and one sample even from three different manufacturers, sold together in one secondary packaging. One further sample contained blisters from the same manufacturer but with two different batch numbers. All these four samples had been obtained from licensed pharmacies. The different blisters of these four purchases were separated and analyzed as separate samples. Thereby, the total numbers of samples of this study increased from 89 to 94. For two of these samples the number of tablets was insufficient for analysis according to USP 39. These two samples were analyzed, but their results were excluded from overall data evaluation and statistical analysis.

In one case, hydrochlorothiazide was requested, but actually a combination product containing hydrochlorothiazide 25 mg and captopril 50mg (Ecazide, BristolMyers Squibb) was obtained. This sample was analyzed according to the methods for hydrochlorothiazide tablets (and found to be compliant with the specifications), but it was excluded from the analysis of prices.

As shown in Table 2, the 94 samples represented 44 different commercial preparations (brands) and a total of 68 different batches. According to the information given on the labels, they were produced by 26 different manufacturers in 12 different countries. Table 3 shows that most of the medicines sold in private pharmacies were from European countries, while most of the medicines sold by informal vendors were produced in African countries. Most of the samples (69 out of 94, i.e. 73%) were generic medicines sold under the international nonproprietary name of their active pharmaceutical ingredient, while 19% were so called branded generics, and only 7 samples (7%) were originator products. All seven originator medicines were obtained in licensed pharmacies. A complete list of all 94 samples, including the results of chemical analysis is shown in S1 Table (Supporting Information).

**Table 2:** Overview of medicine samples collected and analyzed.

Active pharmaceutical ingredient of the sample	Licensed pharmacies	Informal vendors	Total	No. of commercial preparations (brands)	No. of batches
Amoxicillin	6	6	12	6	11
Amoxicillin / clavulanic acid	7	0	7	5	7
Phenoxymethylpenicillin	2	1	3	2	2
Ciprofloxacin	7	6	13	8	11
Doxycycline	8	3	11	5	7
Sulfamethoxazole / trimethoprim	5	1	6	3	5
Metronidazole	6	6	12	4	6
Atenolol	6	1	7	1	3
Furosemide	6	4	10	5	6
Hydrochlorothiazide	3	1	4	3	4
Metformin	6	1	7	1	5
Salbutamol	2	0	2	1	1
Total	64	30	94	44	68

**Table 3:** Comparison of medicine samples from licensed pharmacies and informal vendors.

	Licensed pharmacies	Informal vendors
**Stated origin of medicines**		
- Africa	8 (13%)	19 (63%)
- Asia	11 (17%)	7 (23%)
- Europe	45 (70%)	4 (13%)
**Type of medicine**		
- generic	44 (69%)	25 (83%)
- branded generic	13 (20%)	5 (17%)
- originator	7 (11%)	0 (0%)
**Packaging of purchased sample**		
- primary and secondary packaging (blister strips and cardboard boxes)	55 (86%)	11 (37%)
- only primary packaging (blister strips)	9 (14%)	18 (60%)
- no original packaging (sold in plastic bag)	0 (0%)	1 (3%)

In the six visited pharmacies, on average 10 of the 12 investigated medicines were available (range 8–12 medicines). In the six informal vendors, on average only five medicines were available (range 3–8 medicines). The highest availability was observed for amoxicillin, ciprofloxacin and metronidazole.

S2 Table (Supporting Information) compares the prices of the medicines in licensed pharmacies and informal vendors. Overall, the prices in licensed pharmacies were very similar to those observed previously in the private health care sector in low- and middle- income countries [40]. Prices in informal vendors were cheaper than those in licensed pharmacies by 41%. Further details are given in the legend of S2 Table.

As shown in Table 3, the majority (86%) of samples obtained in private pharmacies were sold with their primary or secondary packaging, i.e. blister stripes and cardboard box. However, the informal vendors sold most of the samples (60%) without secondary packaging. One sample was even dispensed without primary and secondary packaging, i.e. in a plastic bag. Since this sample represented furosemide tablets which are light sensitive, this packaging is in contrast to the requirements specified in the USP 39 monograph. Notably, four further samples of furosemide tablets (two from licensed pharmacies, two from informal vendors) were sold without secondary packaging in clear transparent blisters without light protection, also violating USP39 requirements.

### Expiry dates

None of the samples were expired at the time of purchase. The remaining shelf life of the medicines ranged from 1 to 53 months, with a median of 26 months. 11 samples, produced by Denk Pharma (Germany), Aldo Union (Spain) and Roche (France), had remaining shelf lives of more than 36 months, showing that the manufacturers had decided to state quite long shelf lives for these products.

### Packaging analysis and visual inspection

Inspection of the primary and secondary packaging, of package leaflets and of batch numbers and expiry dates showed no mistakes or inconsistencies which are frequently found in falsified medicines [15, 41]. Therefore, packaging analysis did not indicate the presence of any obviously falsified medicines. Visual inspection of the dosage forms showed a strong discoloration in one sample of doxycycline tablets, depicted in S2 Fig. This sample had contained blisters with two different batch numbers in one secondary packaging, as mentioned above. While the blisters with uniformly colored tablets (exp. date 05.2019) were found to comply with the USP39 specifications, the blister with ten darkened, spotted tablets (exp. date 12.2017) showed extreme deviations from the USP39 specifications (55% of stated content of doxycycline).

### Chemical analysis

As explained above, four samples consisted of blisters of more than one batch and therefore the blisters were analyzed as distinctive samples. For two of the resulting samples (including the ten darkened, spotted doxycycline tablets mentioned above) the number of tablets was too small to perform a complete chemical analysis according to the USP 39. The results of these two samples were therefore excluded from the evaluation of the results, leaving 92 samples for overall data evaluation.

**Fig 1:**
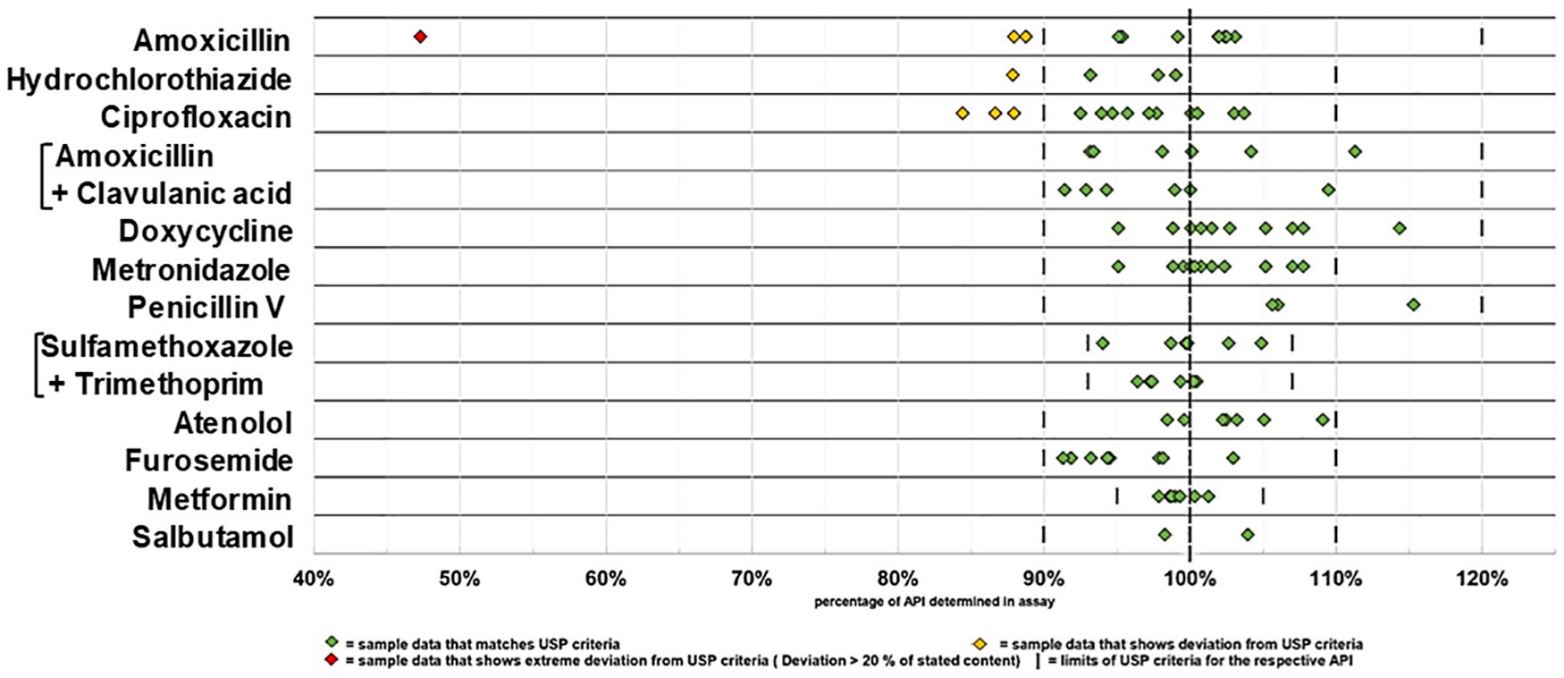
Content of the active pharmaceutical ingredient determined for each sample.

**Fig 2:**
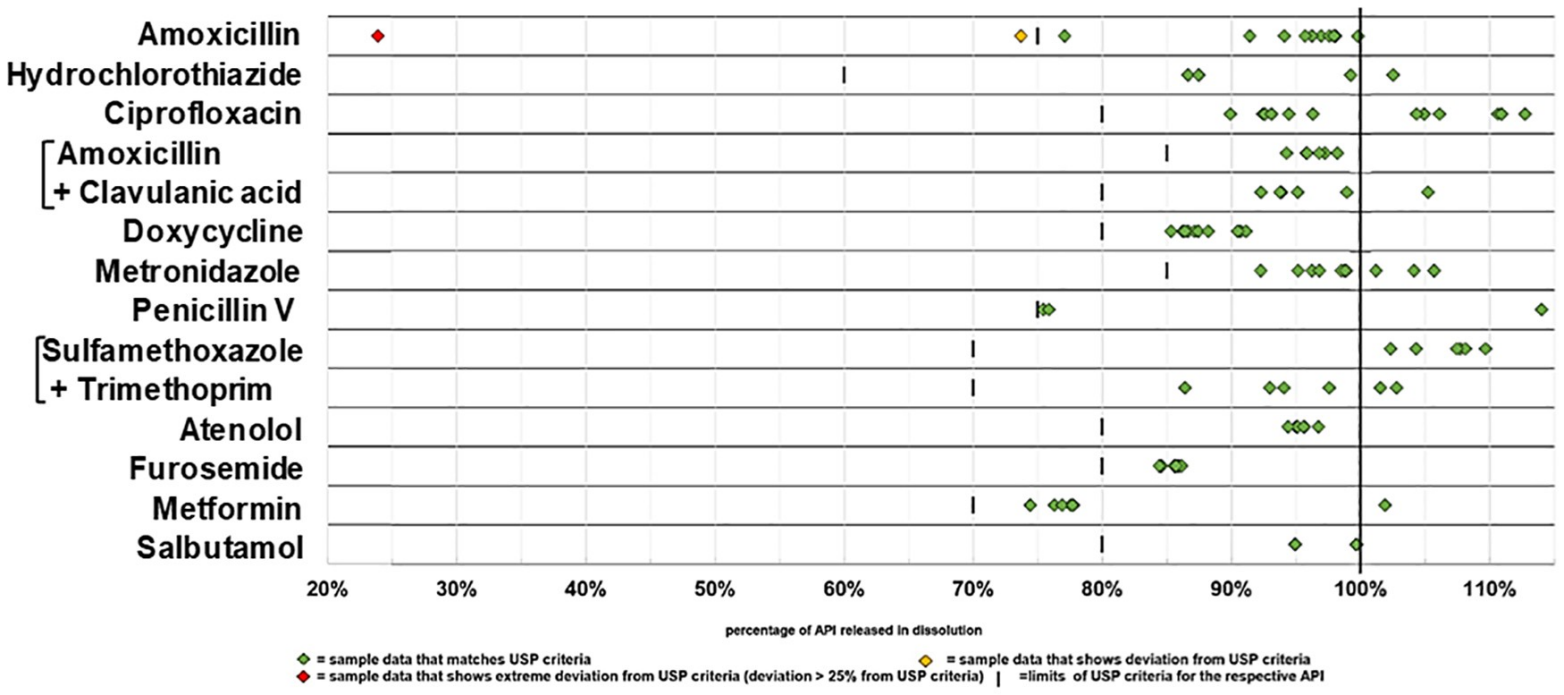
Dissolution of the active pharmaceutical ingredient determined for each sample.

Notably, in none of the 92 samples the stated API was absent, and in only one sample the content of API was lower than 80% of the declared content. Out of the total of 92 samples, 85 (92%, 95%CI = 85–97%) complied with the specifications of USP 39 for both, assay (= content of API) and dissolution. However, 6 samples (7%, 95%CI = 2–14%) showed moderate deviations and 1 samples (1%, 95%CI = 0.03–6%) showed extreme deviations. Table 4 summarizes the results for the 12 tested medicines. Non-compliant samples were observed for amoxicillin, hydrochlorothiazide and ciprofloxacin, but not for any of the other nine investigated medicines. Of the 7 non-compliant samples, 5 failed only in assay, while 2 failed in both criteria. The data summarized in Table 4 are shown in detail in Figs 1 and 2.

**Table 4:** Compliance of medicine samples with the criteria of the United States Pharmacopoeia 39.

API of sample		Non-compliant in assay (number of samples)		Non-compliant in dissolution (number of samples)		Non-compliant, total	
	Total number	Moderate deviation	Extreme deviation	Moderate deviation	Extreme deviation	Number of samples	%
**Amoxicillin**	12	2	1	1	1	3	25%
**Hydrochlorothiazide**	4	1	0	0	0	1	25%
**Ciprofloxacin**	13	3	0	0	0	3	23%
**Amoxicillin/clavulanic acid**	6	0	0	0	0	0	0%
**Doxycycline**	10	0	0	0	0	0	0%
**Metronidazole**	12	0	0	0	0	0	0%
**Phenoxymethylpenicillin**	3	0	0	0	0	0	0%
**Sulfamethoxazole/ Trimethoprim**	6	0	0	0	0	0	0%
**Atenolol**	7	0	0	0	0	0	0%
**Furosemide**	10	0	0	0	0	0	0%
**Metformin**	7	0	0	0	0	0	0%
**Salbutamol**	2	0	0	0	0	0	0%
**Total**	**92**	**6**	**1**	**1**	**1**	**7**	**8%**

The limits for compliance of the USP39, and for extreme deviation according to a WHO publication [11], are explained in the methods section.

Table 5 lists the results of the chemical analysis for each of the 26 stated manufacturers located in 12 countries on three continents. Notably, the highest failure rate was observed in samples deriving from Asia (24% non-compliant). This is markedly higher than the failure rate of the other samples of this study (average 4%) and this difference is statistically significant (p = 0.007). 18 samples had been produced in Togo itself, including 15 from a single Togolese manufacturer. All of the samples produced by that company complied with the USP specifications. This shows an encouraging achievement of international quality standards by this local manufacturer.

**Table 5:** Stated origin and manufacturers of medicines, and compliance with USP 39 criteria.

Stated origin	Stated manufacturer	Total samples	Non-compliant samples	% non-compliant	Extreme deviation	% extreme deviation
**Togo**	Tongmei	15	0		0	
	Sprukfield	3	1		0	
	**total**	**18**	**1**		**0**	
**Benin**	Pharmaquick	**5**	**0**		**0**	
**Ghana**	Letap	**3**	**0**		**0**	
**Nigeria**	Nuel Pharma	**1**	**1**		**0**	
**Africa**		**27**	**2**	**7%**	**0**	**0%**
**India**	Lincoln Pharma	6	1		0	
	Alice	1	0		0	
	Cian	1	1		0	
	CIPLA	1	0		0	
	Fourrts	1	0		0	
	Medopharm	1	0		0	
	**total**	**11**	**2**		**0**	
**China**	Greenfield	1	0		0	
	North China Pharmaceutical	3	1		0	
	Yangzhou NO.3	1	1		1	
	**total**	**5**	**2**		**1**	
**Turkey**	Billim	**1**	**0**		**0**	
**Asia**		**17**	**4**	**24%**	**1**	**6%**
**France**	Baily-Creat	13	0		0	
	GSK-France	3	0		0	
	Roche	1	0		0	
	**total**	**17**	**0**		**0**	
**Germany**	Denk Pharma	16	0		0	
	Philco Pharma	1	0		0	
	**total**	**17**	**0**		**0**	
**Austria**	Sandoz GmbH	**8**	**0**		**0**	
**Spain**	Aldo-Union	2	0		0	
	Novartis	2	1		0	
	**total**	**4**	**1**		**0**	
**Italy**	Bristol Myers Squibb	1	0		0	
	F.I.R.M.A S.p.A	1	0		0	
	**total**	**2**	**0**		**0**	
**Europe**		**48**	**1**	**2%**	**0**	**0%**
**Total**		**92**	**10**	**11%**	**1**	**1%**

**Fig 3:**
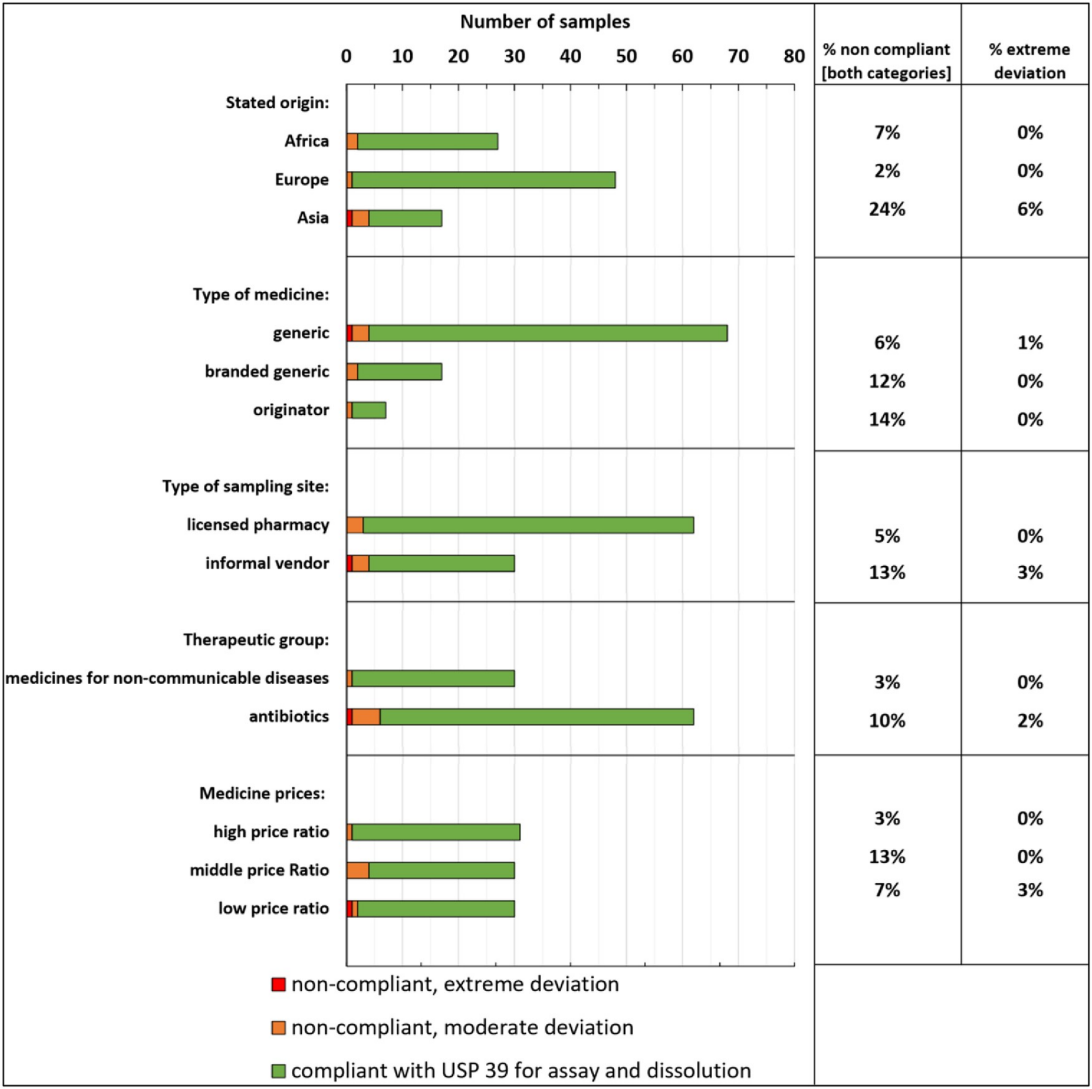
The prevalence of non-compliant medicines grouped according to different criteria.

As mentioned above, this study included 7 originator medicines, all of them obtained in licensed pharmacies and (according to the label information) produced by multinational pharmaceutical companies. One of these seven products failed USP 39 specifications (87.9% of the stated API content determined in assay), while another sample of the same brand with the same expiry date passed all pharmacopoeial tests. We speculate that the poor quality may be the result of inappropriate transport and storage conditions of the individual sample. Fig 3 shows the prevalence of non-compliant medicines, grouped according to different criteria. Generic medicines showed a lower percentage of non-compliant and extremely deviant samples than branded generic medicines or originator products. Medicines obtained from licensed pharmacies showed a lower failure rate than those from informal vendors (5% *versus* 13% non-compliant). However, the difference between the failure rates in licensed pharmacies and informal vendors did not reach statistical significance (p = 0.152).

Quality problems were found both in antibiotics and in medicines for non-communicable diseases (10% and 3% non-compliant, respectively), but the difference between both groups was not statistically significant (p = 0.285).

The frequency of non-compliant samples was not significantly related to the selling price of the medicines (Fig 3). Non-compliant medicines were found in two of the six investigated pharmacies, and in three of the six investigated informal vendors.

### Testing for thermal degradation products of amoxicillin

In order to provide some first evidence whether inappropriate storage and/or transport conditions may have contributed to the substandard quality of some of the investigated medicines, we analyzed several amoxicillin samples for the presence of thermal degradation products of the API. First, reference tablets of amoxicillin were subjected to a forced degradation (four days in a drying oven at 80°C). HPLC analysis according to the method for “related substances” of the USP 39 monograph for amoxicillin trihydrate showed that this treatment led to a reduction of the HPLC peak corresponding to amoxicillin (retention time 7.5 min) by 23%, with the concomitant appearance of a new peak at 48 min (peak area a 230 nm: 2.0% of amoxicillin peak).

Three compliant samples of amoxicillin, and one non-compliant sample showing an insufficient amount of the API, were investigated for the presence of this peak at 48 min. The peak was clearly detected in the non-compliant sample, amounting to 2.0% of the peak area of amoxicillin. The amount of this specific impurity in the non-compliant sample indicates that amoxicillin had undergone some thermal degradation either before or (more likely) after manufacturing of the tablets.

In contrast two compliant samples showed only traces of this peak (< 0.1% of the peak area of amoxicillin), while the third compliant sample did show the peak of the decomposition product (1.8% of the peak area of amoxicillin).

## Discussion

None of the 94 medicine samples collected in this study was obviously falsified, as judged from packaging analysis. All samples contained the declared active pharmaceutical ingredient(s), though in one case less than 50% of the declared amount (Fig 1). A low prevalence of falsified medicines is indeed consistent with results from the current scientific literature [7, 10, 11, 15]. Usually the highest rate of falsified medicines in developing countries is found in antimalarial medicines [15]. In contrast, the present study in Togo focused on antibiotics and medicines for non-communicable diseases. Therefore, the finding that the relatively small number of 94 samples in the present study did not include an obviously falsified sample is not a surprise. This finding does not, however, prove that falsified medicines are absent in Togo. Higher sample numbers and e.g. the inclusion of antimalarials, would most likely show the presence of such medicines [7, 15].

As mentioned above, one sample of amoxicillin capsules contained only 47% of the decared content of the active pharmaceutical ingredient, and the analysis of 10 individual capsules showed a high uniformity of this individual content (SD = 1.72%). This indicates that this sample may have been manufactured as 250mg amoxicillin trihydrate capsules, rather than 500mg as declared on the label. This may represent a deliberate misrepresentation of its composition, i.e. a falsified medicine.

In this study, 8% of the investigated samples did not comply to USP 39 specifications (in assay, dissolution, or both), and 1% showed even an extreme deviation from the pharmacopoeial limits. This prevalence is consistent with recent review published by the WHO which estimated the prevalence of substandard and falsified medicines in low and middle income countries to be 10.5% [7].

It should be noted that a meaningful comparison of failure rates in between different medicine quality surveys in the literature is very difficult. As correctly stated in the recent literature survey by the WHO [7], different studies use different sampling approaches, different analytical techniques, they include or ignore different pharmacopoeial criteria (e.g. dissolution of API) and they use different thresholds for the percentage of the API in order to classify a sample as “within specification” or “out of specification”. In the present study, we investigated the identity, the quantity and dissolution of the API, and followed the analytical methods and the thresholds specified in by the United States Pharmacopoeia 39 (USP 39). The limits for compliance given by the USP 39 are similar to those specified in the International Pharmacopoeia and usually wider than the limits specified by the British Pharmacopoeia.

As noted in the recent literature survey by the WHO [7], most publications of substandard and falsified medicines only state a pass or fail for a chosen threshold, rather than giving actual percentage of the API determined. For the present study, we depicted the actual percentage of the content of API, and of the percentage of the API dissolved in the dissolution test, for all 92 samples in Figs 1 and 2 as well as in the S1 Table. As strikingly obvious from these figures, the results for assay and dissolution are distributed over a wide range, and the percentage of “substandard medicines” depends on the threshold applied in the respective study. In order to improve the comparability of different studies on the quality of medicines, a general reporting of the percentages of the API detected in the individual samples, e.g. as shown in Figs 1 and 2**,** may be useful.

As mentioned in a previous publication by the World Health Organization [11], the observed quality failure rates of medicines cannot always be directly related to therapeutic failures of these medicines. Following that WHO publication, however, we used thresholds for extreme deviations which may be associated with health implications. Within our study, one sample (1%) showed such extreme deviations.

In accordance with our expectations, the failure rate observed in medicines from licensed pharmacies was lower than that from informal vendors, but the difference was smaller than expected and did not reach statistical significance. Notably, the sample showing extreme deviations had been obtained from an informal vendor.

Medicines stated to originate from Asia (i.e. mainly from India and China) showed a significantly higher failure rate than those from Africa and Europe. India certainly has excellent pharmaceutical manufacturers and plays an important role in providing quality generic medicines at low prices to developing countries [41, 42]. However, our study indicates that some manufacturers in India and China supply medicines to Africa which turn out to be substandard. It should be in the interest of regulatory authorities and professional organizations of Asian and African countries to minimize such problems by appropriate regulations and procurement practices, including supplier prequalification schemes.

Several findings reported in the Results section indicate that degradation, possibly due to inappropriate storage and transport conditions, may have contributed to the substandard quality of some of the samples. Notably, the labels of 43 of the 94 samples in this study stated that the products should be stored below 25°C. Yet, none of the investigated licensed pharmacies and informal vendors used an air condition. According to the ICH Quality Guidelines [43], Togo is assigned to climatic zone IV, and long term stability of medicines for use in Togo should be demonstrated at 30°C and 65% relative humidity by the manufacturer. Improvements in regulation and in procurement practice should ensure that only medicines which are compliant in such stability tests are imported to Togo. Health care providers need to pay attention to storage requirements stated on the medicine packages, and either improve the storage conditions in their premises or restrict their selection of medicines to those which do not require storage below 25°C.

Notably, all antibiotics could be purchased without prescription from licensed pharmacies and informal vendors with equal ease. The high availability of antibiotics from informal vendors is worrisome due to the potential of antimicrobial resistance arising from the inappropriate use of antibiotics.

## Limitations of this study

The small size (n = 94 samples) presents a principal limitation of this study which was funded exclusively by intramural funds of Tuebingen University. This study was powered to prove an expected difference of 30 percent in the prevalence of poor quality medicines between pharmacies and informal vendors and was insufficiently powered to prove smaller differences in other sub-group analyses. Alternative approaches within the limits of the available budget may have been a concentration on fewer types of medicines (e.g. just two to four rather than 12 types), or the omission of the time-consuming and expensive dissolution testing in the chemical analysis. However, also these alternatives would have had obvious and strong disadvantages. Another principal limitation of this study is the non-random selection of sampling sites which implies that the results cannot be regarded a representative. In any medicine quality study which includes informal vendors, random sampling of these sites is impossible as there is no reliable list of such illegal facilities. We purposefully identified informal vendors which were reported to be well-stocked, in order to ensure that sufficient numbers of samples of the 12 different medicines could be collected. This may introduce a bias since medicine quality may be different between large and small informal vendors. A list of licensed pharmacies in Togo is published on the internet [27] by a private medicine wholesaler; therefore, an alternative approach would have been a random selection of licensed pharmacies from that (unofficial) list, followed by identification of the nearest informal vendor to that pharmacy. On the other hand, this may have introduced other biases, e.g. in favor of more affluent regions where most licensed pharmacies are located. An additional limitation is that this study sampled medicines only from licensed pharmacies and informal vendors, not from other sectors of the pharmaceutical supply system. And while this study included testing for identity, content and dissolution of the APIs, it did not comprehensively include other criteria such as testing for related substances or content uniformity. As emphasized in a recent WHO publication [7], further studies are required which can provide reliable estimates of the prevalence of substandard and falsified medical products, by product type, geographical distribution and severity of deviation from the pharmacopoeial standards, in Togo as well as in other low- and middle-income countries.

## Supporting information

S1 Table Complete list of all 94 samples. (XLSX)

S2 Table Prices of the medicines in licensed pharmacies and informal vendors. (DOCX)

S1 Fig Map of the sampling sites in the regions Maritime and Plateaux of the Republic of Togo. (DOCX)

S2 Fig Discoloration discovered upon visual inspection of a sample of doxycycline tablets. (DOCX)
