# Supplementary material for: Quality of medicines in southern Togo: Investigation of antibiotics and of medicines for non-communicable diseases from pharmacies and informal vendors
Source: PLoS One. 2018 Nov 29;13(11):e0207911. doi: 10.1371/journal.pone.0207911 (PMC6264819; doi:10.1371/journal.pone.0207911)
Supplement: S2 Table — (DOCX) [file pone.0207911.s002.docx]

**S2 Table: Prices of the medicines in licensed pharmacies and informal vendors**

|  | Median price per dosage form [$] | | median price ratio | |  |
| --- | --- | --- | --- | --- | --- |
| API | formal | Informal | formal | Informal |  |
| Amoxicillin 500 mg | 0.09 | 0.07 | 3.36 | 2.63 |  |
| Amoxicillin / Clavulanicacid 500/125mg | 0.47 |  | 4.96 |  |  |
| Phenoxymethylpenicillin 1000mg | 0.28 | 0.02 (125mg) | 7.57 | 1.36 |  |
| Ciprofloxacin 500mg | 0.18 | 0.06 | 4.74 | 1.53 |  |
| Doxycycline 100mg | 0.07 | 0.04 | 5.04 | 3.07 |  |
| Sulfamethoxazole / Trimethoprim 400/80mg | 0.04 | 0.14 | 3.30 | 12.50 |  |
| Metronidazole 500mg | 0.06 | 0.05 | 6.63 | 3.88 |  |
| Atenolol 50mg | 0.13 | 0.13 | 11.99 | 12.22 |  |
| Furosemide 40mg | 0.07 | 0.04 | 10.72 | 6.70 |  |
| Hydrochlorothiazide 25 / 50mg | 0.06 | 0.03 (50mg) | 14.44 | 6.67 |  |
| Metformin 500mg | 0.08 | 0.08 | 5.28 | 5.25 |  |
| Salbutamol 2mg | 0.12 |  | 49.27 |  |  |
| Overall median price ratio |  |  | 5.28^a^ | 3.18 ^a^ |  |

^a^ median price ratio of all medicines collected

The table shows the median prices per tablet or capsule. Following a standardized method developed by WHO and Health Action International,^1^ those prices were compared to an international reference price, i.e to the median supplier price given in the MSH International Medical Products Price Guide of 2015.^2^ Overall, the resulting median price ratio (MPR) was 5.3 in licensed pharmacies, compared to 3.2 in informal vendors. Therefore, as expected, medicines are more expensive in pharmacies. A United Nations report of 2012^3^ stated that in low and middle income countries the median price ratio in the (formal) private sector was on average 5.3, identical to the figure found for pharmacies in Togo. As expected, the MPR for generic medicines and branded generics (average MPR = 4.1) was lower than that for originator medicines (average MPR= 13.5). Medicines from Africa and Asia (in both cases average MPR = 3.0) were more affordable than those from Europe (average MPR= 7.2). Notably, medicines for non-communicable diseases were sold at much more unfavorable prices (average MPR=10.7) than antibiotics (average MPR=3.1). For the two samples of salbutamol 2mg tablets, sold as branded generics in private pharmacies, the MPR even reached 49.3.

^1^ WHO HAI. Measuring medicines prices, availability, affordability and price components.2008. Available from: http://www.who.int/medicines/areas/access/OMS_Medicine_prices.pdf

^2^ MSH. The International Medical Products Price Guide 2015. Available from: http://mshpriceguide.org/en/home/

^3^ United Nations. Millenium Development Goal 8. The Global Partnership for Development. Making Rhetoric a Reality. MDG Gap Task Force Report 2012. New York. 2012. Available from: http://www.un.org/millenniumgoals/2012_Gap_Report/MDG_2012Gap_Task_Force_report.pdf
