# Supplementary material for: Quality of medicines in southern Togo: Investigation of antibiotics and of medicines for non-communicable diseases from pharmacies and informal vendors
Source: PLoS One. 2018 Nov 29;13(11):e0207911. doi: 10.1371/journal.pone.0207911 (PMC6264819; doi:10.1371/journal.pone.0207911)
Supplement: S1 Fig — (DOCX) [file pone.0207911.s003.docx]

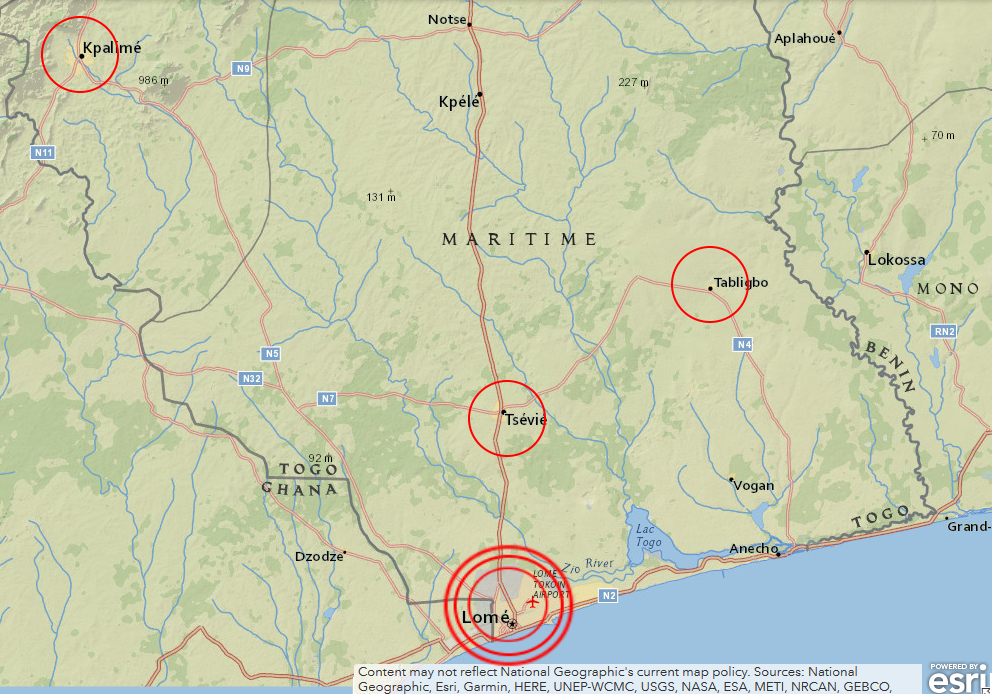


**S 1 Figure:**  **Map of the sampling sites in the regions Maritime and Plateaux of the Republic of Togo** (https://viewer.nationalmap.gov/advanced-viewer/)

In Lomé samples were collected in Lomé Centre and in the suburbs Agoe-nyvie and Agoe-laogope. Furthermore, samples were collected in the towns Tsévié and Tabligbo, 30 km north and 75 km northeast of Lomé, respectively, and in the town of Kpalimé, 120km northwest of Lomé and close to the border to Ghana.
