# Supplementary material for: Quality of medicines in southern Togo: Investigation of antibiotics and of medicines for non-communicable diseases from pharmacies and informal vendors
Source: PLoS One. 2018 Nov 29;13(11):e0207911. doi: 10.1371/journal.pone.0207911 (PMC6264819; doi:10.1371/journal.pone.0207911)
Supplement: S2 Fig — (DOCX) [file pone.0207911.s004.docx]

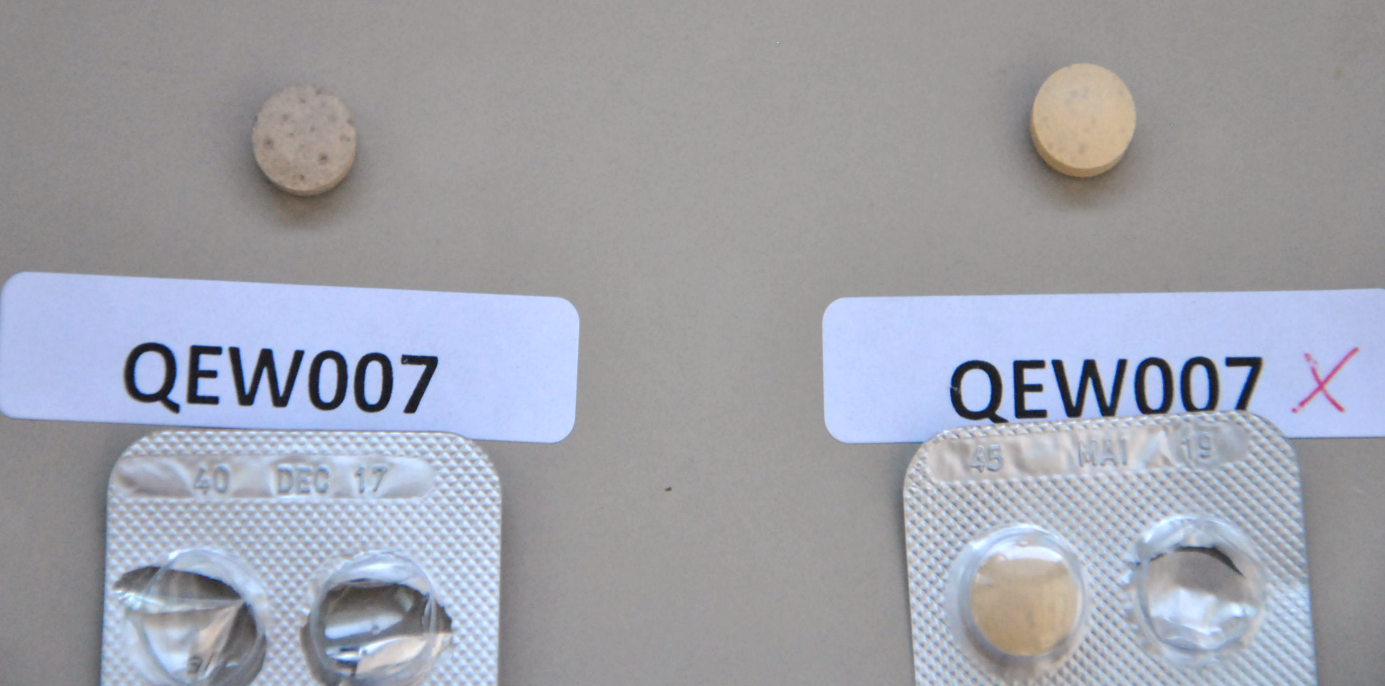


**S2 Figure: Discoloration discovered upon visual inspection of a sample of doxycycline tablets**

This sample of doxycycline 100mg tablets (Bailly-Creat, France) contained blisters with two different batch numbers in one secondary packaging. While the blisters with the uniformly colored tablets (batch no. 45; exp. date 05.2019) depicted on the right side (QEW007X) were found to comply with the USP39 specifications, the blisters with the darkened, spotted tablets (batch no. 40; exp. date 12.2017) depicted on the left side (QEW007) showed extreme deviations from the USP39 specifications both in the assay (58% of stated content of doxycycline) and in dissolution. Since QEW007 consisted of only one blister of 10 tablets, which was lower than the amount required by the USP 39 monographs, the results for this sample were excluded from the overall data analysis.
